# The neo-aortic valve in patients with hypoplastic left heart syndrome is largely preserved: a serial follow-up CMR study

**DOI:** 10.3389/fcvm.2024.1466982

**Published:** 2024-11-05

**Authors:** Abigail Burleigh, Dominik Daniel Gabbert, Yujiro Ide, Inga Voges

**Affiliations:** ^1^ Department of Congenital Heart Disease and Paediatric Cardiology, University Hospital Schleswig-Holstein, Kiel, Germany; ^2^ German Center for Cardiovascular Research (DZHK), Partner Site Hamburg/Lübeck/Kiel, Hamburg, Germany

**Keywords:** hypoplastic left heart syndrome, neo-aortic valve, cardiovascular magnetic resonance, ventricular function, neo-aortic regurgitation

## Abstract

**Background:**

In hypoplastic left heart syndrome (HLHS) patients, neo-aortic valve regurgitation can negatively impact right ventricular (RV) function. We assessed neo-aortic valve function and RV volumetric parameters by analysing serial cardiovascular magnetic resonance (CMR) studies in HLHS patients after completion of total cavopulmonary connection (TCPC).

**Methods:**

Consecutive CMR examinations of 80 patients (female: 22) with two (*n* = 80) or three (*n* = 45) examinations each were retrospectively analysed. RV volumetry was performed using short-axis cine images. RV end-diastolic and end-systolic volumes normalised to body surface area (BSA, RVEDVi, RVESVi), ejection fraction (RVEF) and stroke volume (RVSV) were measured. Neo-aortic flow, regurgitant fraction (RF) and peak velocity were quantified from phase-contrast cine images.

**Results:**

Median neo-aortic regurgitation was mild at all three examinations (RF <20%) and there was no significant increase in RF over time (*p* > 0.05). None of the patients had significant neo-aortic valve stenosis (peak velocity >3 m/s). RF correlated with RVESVi and RVEF at the second examination. At the third examination, RF correlated with RVESVi and RVEDVi even in patients with RF <15% (RVESVi: *r* = 0.40, *p* = 0.001; RVEDVi: *r* = 0.34, *p* = 0.031).

**Conclusion:**

Assessment of serial CMR studies in HLHS patients after TCPC completion demonstrates a preserved neo-aortic valve function. Nevertheless, thorough follow-up is mandatory as even mild neo-aortic dysfunction might impact RV size and function over a longer term.

## 1 Introduction

Hypoplastic left heart syndrome (HLHS) has a reported prevalence of ∼1.5/10.000 live births and is one of the most severe congenital heart defects (1). Characterised by a complex spectrum of cardiac anomalies, including a substantial hypoplasia of the left ventricle, its associated structures and the ascending aorta, it is fatal if left untreated (2). Palliation is achieved in three stages, with the total cavopulmonary connection (TCPC) being the final stage (3). During palliation, a neo-aorta and neo-aortic valve are constructed from the hypoplastic native aorta and the native proximal pulmonary artery (4). The function of the neo-aortic valve after completion of the total cavopulmonary connection, i.e., in patients with Fontan circulation, is of clinical interest and relevance. Its dysfunction, namely neo-aortic valve regurgitation, can negatively impact right ventricular (RV) function, thus forming an important sequela (5, 6). Despite the neo-aortic valve being a key-structure in the palliated heart's anatomy, research on its regurgitation is still sparse.

We hypothesized that neo-aortic valve function is preserved in the longer-term in the majority of patients.

Therefore, the aim of this cardiovascular magnetic resonance (CMR) study was (1) to assess the neo-aortic valve function during serial follow-up and (2) to examine the impact of neo-aortic valve regurgitation on RV volumes and systolic function.

## 2 Methods

### 2.1 Patients

Patients with HLHS who were under regular follow-up at our institution were included. Inclusion criteria were (1) a minimum of two CMR examinations after completion of the total cavopulmonary connection, (2) availability of short axis cine images and two-dimensional (2D) phase-contrast images of the ascending aorta and (3) the examinations had to be of sufficient quality. Patients with only one, incomplete or insufficient CMR examinations as well as patients with contraindications for CMR were disregarded.

Informed consent was obtained from the parents or guardians of the children enrolled into the study. The study protocol conforms to the ethical guidelines of the 1975 Declaration of Helsinki as reflected in *a priori* approval by the institution's human research committee (ID Nr.: D503/20, date of approval 12th June 2021 and approval of amendment 18th October 2021).

### 2.2 CMR analysis

CMR studies were performed using a 1.5 Tesla (T) or 3 T scanner.

Image postprocessing was carried out using commercially available software (cvi42 for Cardiovascular MRI, Circle Cardiovascular Imaging, Calgary, Canada; Medis Suite Solutions, Medical Imaging Software, Leiden, the Netherlands). Manual tracing of endo- and epicardial contours on short axis cine images was performed to assess RV end-diastolic, end-systolic and stroke volumes (RVEDV, RVESV, RVSV), as well as RV ejection fraction. To achieve comparability, RV volumes were indexed to body surface area (BSA, RVEDVi, RVESVi, RVSVi). 2D phase-contrast images in the ascending aorta were used to measure neo-aortic net flow, regurgitant fraction (RF) and peak velocity. The degree of neo-aortic regurgitation was classified as follows: (1) mild (RF 5%–20%), (2) moderate (RF 20%–40%) and (3) severe (RF >40%) (7). Moderate neo-aortic stenosis was defined as a peak velocity in the ascending aorta above 3 m/s.

### 2.3 Statistical analysis

Analysis was performed using MedCalc® (version 22.016, MedCalc Software Ltd, Belgium). Normal distribution was assessed using the Shapiro-Wilk test. Normally distributed data are shown as mean and standard deviation (SD), non-normally distributed data are shown as median and 1st and 3rd quartiles (IQR) or median and range. The difference in aortic RF from the first to the second examination and the difference between the first/second and the third examination was analysed using a Wilcoxon signed-rank test. The test hypothesis was that there are no changes over time. Spearman's coefficient of rank correlation for non-normally distributed data was used to examine correlations between RV volumetric and neo-aortic flow parameters.

## 3 Results

Patient demographics and results from CMR examinations are shown in Table 1.

**Table 1:** Patient characteristics and CMR results.

Parameters	1st examination (*n* = 80)	2nd examination (*n* = 80)	*p*-value*, comparison between 1st and 2nd examination	3rd examination (*n* = 42)	*p*-value*, comparison between 2nd and 3rd examination
Age (y)	4.4 [3.8; 6.0]	10.0 [8.9; 10.6]	<0.001	15.2 [13.5; 16.5]	<0.001
Weight (kg)	16.7 [15.0; 18.8]	30.0 [25.5; 37.0]	<0.001	51.0 [43.7; 65.8]	<0.001
Height (cm)	104.0 [99.0; 111.0]	137.0 [129.0; 146.5]	<0.001	162.6 [153.8; 172.3]	<0.001
BSA (m^2^)	0.7 [0.6; 0.8]	1.1 [0.96; 1.2]	<0.001	1.5 [1.4; 1.7]	<0.001
RVEDV (ml)	61.9 [51.4; 72.9]	102.3 [82.8; 127.2]	<0.001	165.4 [132.8; 198.8]	<0.001
RVEDVi (ml/m^2^)	86.1 [74.2; 98.5]	90.4 [74.3; 110.2]	0.04	107.4 [94.1; 120.9]	<0.001
RVESV (ml)	27.8 [20.9; 34.8]	48.0 [36.4; 62.4]	<0.001	80.0 [62.1; 105.7]	<0.001
RVESVi (ml/m^2^)	37.5 [29.7; 46.8]	42.6 [34.3; 55.5]	<0.001	52.0 [42.5; 68.3]	<0.001
RVSV (ml)	31.8 [29.5; 37.9]	51.4 [45.0; 67.4]	<0.001	77.7 [64.2; 95.4]	<0.001
RVSVi (ml/m^2^)	47.4 [42.2; 53.3]	49.6 [42.7; 58.5]	0.09	53.3 [46.0; 52.2]	0.001
RVEF (%)	55.5 [50.3; 59.7]	54.2 [48.8; 59.0]	0.03	50.6 [54.8; 56.4]	<0.001
AAo net flow (l/min)	2.1 [1.8; 2.5]	3.2 [2.6; 3.8]	<0.001	4.7 [4.1; 5.5]	<0.001
AAo net flow (l/min/m^2^)	3.0 [2.7; 3.5]	2.9 [2.5; 3.4]	0.15	3.0 [2.5; 3.6]	0.12
Ao RF (%)	3.6 [2.4; 5.6]	3.3 [2.0; 6.7]	0.89	4.9 [2.9; 7.1]	0.11
AAo peak velocity (m/s)	0.8 [0.7; 0.9]	0.9 [0.8; 1.0]	<0.001	1.0 [0.9; 1.2]	<0.001

AAo, ascending aorta; Ao RF, aortic regurgitant fraction; BSA, body surface area; RVEDV, right ventricular end-diastolic volume; RVEDVi, indexed right ventricular end-diastolic volume; RVEF, right ventricular ejection fraction; RVESV, right ventricular end-systolic volume; RVESVi, indexed right ventricular end-systolic volume; RVSV, right ventricular stroke volume; RVSVi, indexed right ventricular stroke volume; y, years.

Data are presented as median and IQR.

* Wilcoxon signed-rank test.

80 HLHS patients (22 females) were included. Median age at TCPC was 2.6 years (y) (range 1.4–5.4 y). Every patient in the cohort had two CMR examinations and 42 patients had three examinations. The first CMR examination was performed at a median age of 4.4 y [3.8; 6.0]. The median interval between the first and second CMR examination was 5.3 y [4.8; 6.0], and 10.1 y [8.8; 11.6] between the first and third examination.

Median neo-aortic regurgitation was below 5% at all three time points and there was no significant increase in RF over time (Table 1; *p* values ranged from 0.1 to 0.9). Only two patients had more than mild neo-aortic regurgitation (RF >20%). Neo-aortic net flow normalised to BSA did not change significantly across examinations (Table 1) either. None of the patients had significant neo-aortic valve stenosis defined as a peak velocity in the ascending aorta of >3 m/s.

**Figure 1:**
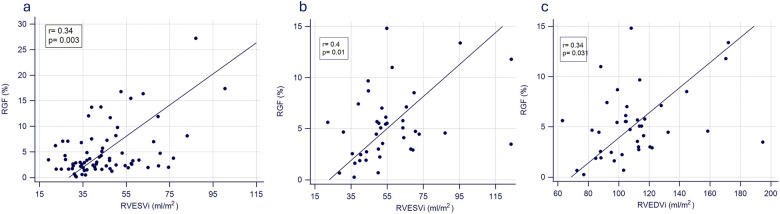
Spearman rank correlation at the second **(a)** and third **(b, c)** examination.

Spearman rank correlation showed that RF correlated with RVESVi and RVEF at the second examination (RVESVi: *r* = 0.34, *p* = 0.003, Figure 1; RVEF: *r* = 0.24, *p* = 0.036). Similarly, RF correlated significantly with RVESVi (*r* = 0.47, *p* = 0.002) as well as with RVEDVi (*r* = 0.43, *p* = 0.005) at the third examination. To check whether mild neo-aortic regurgitation has an impact on RV volumes, associations between RF and RV volumes in patients with RF <15% were analysed. In these patients, significant positive correlations between RF and RVESVi (*r* = 0.40, *p* = 0.001) as well as RVEDVi (*r* = 0.34, *p* = 0.031) at the third examination were found.

## 4 Discussion

Neo-aortic valve function plays a crucial role for HLHS patients post-Norwood operation. By analysing serial CMR studies covering a median time period of 10.3 y between the first and third examination, it could be shown that the neo-aortic function is largely preserved and only a few patients have more than mild neo-aortic regurgitation.

Only few previous studies have analysed neo-aortic regurgitation over time. In an earlier study from 2003, Cohen et al. used serial echocardiograms to assess the degree of regurgitation over time in a cohort of 53 HLHS patients. Although they found an increase of neo-aortic regurgitation over time, similarly to our results only very few patients (*n* = 3) had more than mild neo-aortic regurgitation (6). In a smaller cohort, another group assessed whether the type of initial palliation in HLHS patients impacts the degree of neo-aortic regurgitation. They could not find a difference between the palliation groups and the degree of neo-aortic regurgitation assessed by echocardiography was mild in all three groups (5).

Cohen et al. could show progressive neo-aortic dilatation by echocardiography that was related to the presence of neo-aortic regurgitation (6), whereas Umezu et al. did not show a significant neo-aortic valve *z* score change over time (5). From previous studies in patients after arterial switch operation and patients after Ross procedure, it is know that both the neo-aortic valve (previous pulmonary valve) and neo-aortic root dilates and that this is associated with neo-aortic regurgitation (8–10). Reasons for neo-aortic dilatation are most likely multifactorial including abnormal arterial wall properties (11, 12). However, this has not been well studied in HLHS patients. In the present study, neo-aortic dimensions were not assessed and future studies that pay attention to potential factors that can impact neo-aortic size are needed.

Apart from neo-aortic dilatation, other reasons for neo-aortic regurgitation in HLHS patients might include surgical strategies (e.g., material used for the Norwood operation or banding of the pulmonary trunk prior to the Norwood operation) (5, 13, 14).

This study used CMR data to assess the function of the neo-aortic valve together with the size and systolic function of the systemic right ventricle. A benefit of CMR is that it allows an accurate and reproducible assessment of the degree of aortic regurgitation (15) as well as of ventricular volumes and function (16). Correlations between the measured RV volumes and the neo-aortic RF showed a positive relationship during follow-up that was present even in patients with a neo-aortic RF <15%, suggesting that small amounts of regurgitation can negatively impact ventricular volumes. As the size of the systemic ventricle matters in terms of clinical outcome (17, 18), this result seems of importance. It has been shown that increased ventricular volumes measured by CMR are associated with reduced cardiopulmonary fitness (19), are predictors of death and heart transplant late after Fontan completion (18) and that the extent of increase in end-diastolic volume poses a higher risk for death or a need of heart transplantation (17). Surveillance imaging using CMR, as recommended by the American Heart Association (20), should pay attention to this especially when patients get older. The follow up should be performed in specialised centers paying careful attention to neo-aortic valve function and systemic RV dimensions and function with more frequent CMR follow ups if there is a suspicion of increasing neo-aortic regurgitation and RV size. As reasons for neo-aortic regurgitation are not fully understood, future studies potentially using modern imaging techniques are needed.

### 4.1 Limitations

This is a retrospective study and the number of patients who had a third CMR examination was lower than those who had two CMR studies. In our department, patients receive a CMR examination one year after Fontan completion and then every 5 years in childhood and every 3 years in adulthood. The reason for the lower number of cases at the time of the third examination is therefore mainly due to the relatively young age of the patients. Future studies should examine larger cohorts and longer follow-up times with standardized protocols. Furthermore, neo-aortic root size as a parameter that might influence neo-aortic regurgitation was not measured.

## 5 Conclusion

By analysing serial CMR examinations, it could be shown that neo-aortic valve function in HLHS patients after TCPC completion is overall preserved. However, the presence of even mild neo-aortic regurgitation negatively impacts RV size and function. Therefore, thorough life-long follow-up seems mandatory.

## Data Availability

The raw data supporting the conclusions of this article will be made available by the authors, without undue reservation.
